# Congenital anomalies and predisposition to severe COVID-19 among pediatric patients in the United States

**DOI:** 10.1038/s41390-024-03076-9

**Published:** 2024-02-16

**Authors:** Laura F. Goodman, Peter T. Yu, Yigit Guner, Saeed Awan, Akhil Mohan, Kevin Ge, Mathew Chandy, Mario Sánchez, Louis Ehwerhemuepha

**Affiliations:** 1 Children’s Hospital of Orange County, Orange, CA USA; 2 University of California-Irvine Department of Surgery, Orange, CA USA; 3 University of Arizona, Tucson, AZ USA; 4 Emory University, 201 Dowman Dr, Atlanta, GA USA; 5 University of Connecticut, Storrs, CT USA; 6 Chapman University, School of Computational and Data Sciences, Orange, CA USA

## Abstract

**Background and objective:**

Congenital heart defects are known to be associated with increased odds of severe COVID-19. Congenital anomalies affecting other body systems may also be associated with poor outcomes. This study is an exhaustive assessment of congenital anomalies and odds of severe COVID-19 in pediatric patients.

**Methods:**

Data were retrieved from the COVID-19 dataset of Cerner® Real-World Data for encounters from March 2020 to February 2022. Prior to matching, the data consisted of 664,523 patients less than 18 years old and 927,805 corresponding encounters with COVID-19 from 117 health systems across the United States. One-to-one propensity score matching was performed, and a cumulative link mixed-effects model with random intercepts for health system and patients was built to assess corresponding associations.

**Results:**

All congenital anomalies were associated with worse COVID-19 outcomes, with the strongest association observed for cardiovascular anomalies (odds ratio [OR], 3.84; 95% CI, 3.63–4.06) and the weakest association observed for anomalies affecting the eye/ear/face/neck (OR, 1.16; 95% CI, 1.03–1.31).

**Conclusions and relevance:**

Congenital anomalies are associated with greater odds of experiencing severe symptoms of COVID-19. In addition to congenital heart defects, all other birth defects may increase the odds for more severe COVID-19.

**Impact:**

- All congenital anomalies are associated with increased odds of severe COVID-19.
- This study is the largest and among the first to investigate birth defects across all body systems.
- The multicenter large data and analysis demonstrate the increased odds of severe COVID19 in pediatric patients with congenital anomalies affecting any body system. These data demonstrate that all children with birth defects are at increased odds of more severe COVID-19, not only those with heart defects. This should be taken into consideration when optimizing prevention and intervention resources within a hospital.

## Introduction

Congenital anomalies are structural or functional anomalies present at birth^1,2^ that can affect any body system including the respiratory, cardiovascular, gastrointestinal, and neurologic systems. The World Health Organization estimates that 6% of children worldwide have a congenital anomaly,^2^ while the Centers for Disease Control and Prevention (CDC) estimate the rate to be 3% among US newborns.^3^ In the US, 20% of children affected by congenital anomalies die in infancy, and such anomalies are the most common cause of mortality in the first year of life.^4,5^ In some cases, these congenital anomalies, such as those affecting the cardiovascular system, Trisomy 21, and gastroschisis,^6^ are increasing in incidence annually.^7^

The SARS-CoV-2 virus or Coronavirus 2019 (COVID-19) pandemic has exacerbated the health burden of children with certain preexisting conditions.^8–12^ Previous work by our group showed a strong association between congenital heart disease (CHD) and COVID-19 severity in children,^8^ but there is a dearth of large multicenter studies on congenital anomalies affecting other body systems. Therefore, there is a need to investigate the influence of congenital anomalies on the clinical course of SARS-CoV-2 infection in order to optimize prevention efforts and allocate hospital resources to those at increased risk for severe COVID-19.^8^

In this study, we exhaustively assessed the association between congenital anomalies and severity of COVID-19 among pediatric patients. We hypothesized that, in addition to those affecting the cardiovascular system^8^, other congenital anomalies may be associated with worse outcomes, and that there would be significant differences in the magnitude of association according to the physiologic system affected.

## Methods

This retrospective cohort study was approved by the Children’s Hospital of Orange County Institutional Review Board (IRB #2008107).

### Data sources

Cerner® Real-World Data (CRWD)—a large multicenter electronic health records (EHR) database—was used for this study. As of March 2022, the CRWD system houses data from 120 health systems and over 1.4 billion encounters from all care settings in the United States. It is a clinical data warehouse powered by Cerner’s EHR-neutral and insights platform (HealtheIntent).^13,14^ HealtheIntent retrieves data from the EHR of individual health systems. This data is combined across health systems, de-identified, encrypted, and secured in compliance with the Health Insurance Portability and Accountability Act of 1996 privacy regulation.^13,14^

### Patients and variables

In this study, we used the COVID-19 dataset from CRWD and retrieved records for COVID-19 encounters that occurred between March 1, 2020 and February 28, 2022 for patients less than 18 years of age. Congenital anomalies were obtained by searching all relevant diagnosis codes in the database. International Statistical Classification of Disease, Version 10, Codes (ICD-10-CM) were used to identify congenital anomalies affecting organs or body systems such as the nervous system (Q00-Q07), eye, ear, face and neck (Q10-Q18), circulatory system (Q20-Q28), respiratory system (Q30-Q34), cleft lip and cleft palate (Q35-Q37), other digestive system organs (Q38-Q45), genital organs (Q50-Q56), urinary system (Q60-Q64), musculoskeletal system (Q65-Q79), other congenital malformations such as congenital ichthyosis, epidermolysis bullosa, phakomatoses, congenital malformation of the spleen, etc.^15^ (Q80-Q89), and chromosomal abnormalities (Q90-Q99). These large groups of congenital anomalies, based on the organ/body systems affected, were selected, a priori, to make the study feasible and as a guide for investigation of more specific conditions encompassed within them. All body systems for which a patient may have a congenital malformation were accounted for. Data on patient diagnoses were provided in the database from the year 2015. If the diagnosis was reported and resolved prior to 2015, the patient may not have been designated as having a congenital anomaly.

Complex chronic conditions in pediatric patients have been previously classified using both diagnosis and procedure codes into broad classes of chronic conditions.^16^ We adopted the definitions published by Feudtner et al. (2014) for pediatric chronic conditions encompassing neurologic/neuromuscular, cardiovascular, respiratory, renal/urologic, gastrointestinal, hematologic/immunologic, metabolic, prematurity/neonatal, and devices and transplants.^16^

We retrieved important demographic and related variables to be controlled for in a multivariable model including sex, race, age at encounter, and health insurance payor. This model also controlled for number of non-congenital chronic conditions affecting the neurologic/neuromuscular, cardiovascular, respiratory, renal/urologic, gastrointestinal, hematologic/immunologic, and metabolic body systems, as well as malignancy, prematurity/neonatal chronic conditions,^16^ and obesity status (determined using the 95^th^ percentile body mass index (BMI) threshold, respective to age). If BMI was not reported, the presence of a diagnosis code was used to determine obesity status.^8^

COVID-19 severity was defined as an ordinal variable by the level of respiratory support administered to the patient during the hospital encounter.^8,17,18^ Patients who required invasive oxygen therapy (such as mechanical ventilation and extracorporeal membrane oxygenation) or died were defined as having had severe COVID-19. Patients discharged alive who received noninvasive oxygen therapy (such as bilevel positive airway pressure, continuous positive airway pressure, nasal cannula, or high-flow nasal cannula) were defined as having had moderate COVID-19. All other patients, those  who were discharged alive and did not receive oxygen therapy, were defined as having had asymptomatic or mild COVID-19. The use of oxygen supplementation as an indicator of COVID-19 severity was selected based on existing literature.^8,17,18^

### Statistical analysis

Matching between patients with congenital anomalies and their peers with no congenital anomalies was carried out to reduce both measured and unmeasured bias. This was achieved using one-to-one propensity score matching on age at encounter and number of other classes of chronic conditions with exact matching on race, sex, month of encounter, and health system. Consequently, matching was performed at the encounter level such that encounters occurring during the same month (or period) of the pandemic were matched to reduce unmeasured bias that may be associated with differences in variants of SARS-CoV-2, availability of vaccines and vaccination status, changes in treatment, and changes in public health policies. Furthermore, matching on health system was performed to reduce unmeasured bias associated with differences in local treatment patterns, local surges, and community response to the virus. Caliper width on the logit of the propensity scores was set to 0.2 standard deviations of the logit—a value based on extensive series of Monte Carlo simulations.^19^ The goal of matching was to reduce baseline differences between children with congenital anomalies and their peers with no anomalies.

A cumulative link mixed-effects model (based on the proportion odds assumption) was used for modeling because COVID-19 severity was defined as an ordinal variable, patients were clustered within their respective health systems, and some patients had more than one encounter (reinfection) during the study period. Random intercepts for health systems and patients were introduced to account for correlations within health systems and cases of multiple encounters per patient. A “base model” was constructed on baseline variables including age at encounter, sex, race, payor, number of chronic conditions, and obesity. A “full model” was constructed using variables from the “base model” in addition to congenital and chronic conditions under test of hypothesis. An omnibus test was performed to determine whether congenital anomalies or chronic diagnoses were significant at an alpha of 0.05. Analyses were conducted using Spark and R, including the “ordinal” package for cumulative link mixed models.^20–22^

## Results

In total, there were 927,805 pediatric patient encounters with COVID-19 from March 2020 to February 2022. The unmatched cohort comprised 846,758 (91.2%) patients who were asymptomatic or had mild COVID-19, 60,299 (6.5%) patients who experienced moderate COVID-19, and 20,748 (2.2%) patients who experienced severe COVID-19 symptoms. The matched cohort comprised 85,649 (80.0%) patients who were asymptomatic or had mild COVID-19; 14,317 (13.4%) patients with moderate COVID-19; and 7014 (6.6%) patients with severe COVID-19. Refer to Table 1 for summary statistics on the matched cohort and to the supplemental material for summary statistics on the full/unmatched data. (Table 2)

**Table 1:** Summary statistics by COVID-19 severity.

Variables	COVID-19 severity		
	Mild (*n* = 85,649)	Moderate (*n* = 14,317)	Severe (*n* = 7014)
Age (median, IQR)	2 (0, 9)	4 (1, 12)	2 (0, 11)
Sex			
Female	37,075 (43%)	5871 (41%)	2964 (42%)
Male	48,502 (57%)	8436 (59%)	4048 (58%)
Unknown	72 (<0.1%)	10 (<0.1%)	2 (<0.1%)
Race			
White	52,872 (62%)	9397 (66%)	4283 (61%)
American Indian/Alaska Native	487 (0.6%)	90 (0.6%)	33 (0.5%)
Asian or Pacific Islander	1680 (2.0%)	349 (2.4%)	161 (2.3%)
Black or African American	15,583 (18%)	2059 (14%)	1202 (17%)
Mixed racial group	772 (0.9%)	156 (1.1%)	28 (0.4%)
Other racial group	9337 (11%)	1399 (9.8%)	714 (10%)
Unknown racial group	4918 (5.7%)	867 (6.1%)	593 (8.5%)
Payor			
Commercial	22,046 (26%)	5089 (36%)	2219 (32%)
Governmental	28,295 (33%)	4576 (32%)	2057 (29%)
Other or unknown	34,359 (40%)	4531 (32%)	2685 (38%)
Self-Pay	949 (1.1%)	121 (0.8%)	53 (0.8%)
Encounter type			
Admitted for observation	8140 (9.5%)	3137 (22%)	317 (4.5%)
Emergency	47,577 (56%)	1067 (7.5%)	171 (2.4%)
Inpatient	16,690 (19%)	10,053 (70%)	6526 (93%)
Urgent care encounter	13,242 (15%)	60 (0.4%)	0 (0%)
Obesity	9128 (11%)	1979 (14%)	860 (12%)
Non-congenital chronic conditions (Median, IQR)	0 (0, 1)	1 (0, 2)	1 (0, 2)
Congenital anomalies by body system			
Neurologic	3946 (4.6%)	1449 (10%)	968 (14%)
Eye, ear, face, and neck	2253 (2.6%)	440 (3.1%)	324 (4.6%)
Circulatory	9013 (11%)	2702 (19%)	2676 (38%)
Respiratory	2366 (2.8%)	814 (5.7%)	710 (10%)
Cleft lip/palate	801 (0.9%)	369 (2.6%)	223 (3.2%)
Other digestive systems	3413 (4.0%)	1350 (9.4%)	660 (9.4%)
Genitourinary	6179 (7.2%)	1359 (9.5%)	658 (9.4%)
Musculoskeletal	10,685 (12%)	2471 (17%)	1338 (19%)
Other congenital anomalies	6038 (7.0%)	1450 (10%)	968 (14%)
Chromosomal	4917 (5.7%)	1350 (9.4%)	984 (14%)
Medications			
Remdesivir	62 (<0.1%)	145 (1.0%)	184 (2.6%)
COVID-19 convalescent plasma	3 (<0.1%)	6 (<0.1%)	3 (<0.1%)
Dexamethasone	6591 (7.7%)	5337 (37%)	3095 (44%)
Heparin	799 (0.9%)	1209 (8.4%)	2234 (31.9%)
Immunoglobin therapy	236 (0.3%)	85 (0.6%)	113 (1.6%)
Methylprednisolone	728 (0.8%)	888 (6.2%)	1,207 (17%)
Rituximab	50 (<0.1%)	36 (0.3%)	9 (0.1%)
Tocilizumab	16 (<0.1%)	16 (0.1%)	31 (0.4%)
Aspirin	810 (0.9%)	735 (5.1%)	899 (13%)
Lopinavir/ritonavir	0 (0%)	1 (<0.1%)	0 (0%)

**Table 2:** Cumulative link mixed-effects model for congenital anomalies.

Variables	Odds ratios	*p* value
Congenital anomalies		
Circulatory	3.84 (3.63, 4.06)	<0.001
Cleft lip/palate	2.87 (2.48, 3.32)	<0.001
Other digestive systems	2.62 (2.42, 2.84)	<0.001
Respiratory	2.24 (2.04, 2.46)	<0.001
Neurologic	2.15 (1.99, 2.32)	<0.001
Musculoskeletal	1.68 (1.58, 1.77)	<0.001
Chromosomal	1.54 (1.43, 1.66)	<0.001
Genitourinary	1.32 (1.22, 1.41)	<0.001
Other congenital anomalies	1.23 (1.15, 1.32)	<0.001
Congenital anomalies of the eye, ear, face and neck	1.16 (1.03, 1.31)	0.01
Variables controlled for^a^		
Age (years)	1.02 (1.02, 1.03)	<0.001
Sex (Reference, Female)		
Male	1.17 (1.13, 1.22)	<0.001
Other/unknown	0.93 (0.44, 1.94)	0.84
Race (Reference, White)		
American Indian or Alaska Native	0.94 (0.72, 1.23)	0.65
Asian or Pacific Islander	1.06 (0.93, 1.21)	0.37
Black or African American	0.75 (0.70, 0.79)	<0.001
Mixed racial group	0.76 (0.62, 0.95)	0.01
Other racial group	0.77 (0.71, 0.83)	<0.001
Unknown racial group	1.06 (0.98, 1.16)	0.15
Payor (Reference, Commercial)		
Governmental	0.78 (0.73, 0.82)	<0.001
Other/unknown	0.64 (0.61, 0.68)	<0.001
Self-pay	0.70 (0.58, 0.86)	0.001
Obesity	1.21 (1.14, 1.29)	<0.001
Number of other chronic conditions	1.26 (1.25, 1.28)	<0.001

^a^These variables also made up the base model. Omnibus test was between the base model and a full model containing all variables in this table. Statistical significance was assessed using the likelihood ratio test *p* value of the omnibus test.

**Fig. 1:**
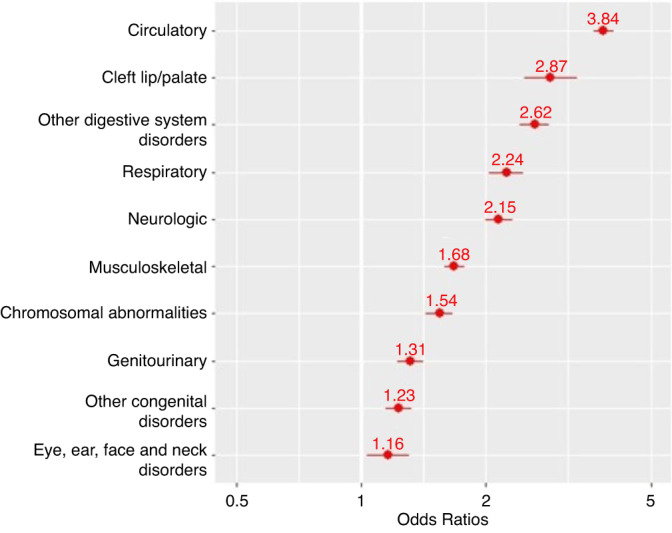
Association between congenital anomalies and COVID-19 severity.

A likelihood ratio (omnibus) test, between the full and base models, suggested that congenital anomalies were significantly associated with the severity of COVID-19 (*p* < 0.001). Congenital anomalies observed (in decreasing magnitude of odds ratios) were: circulatory disorders, with 284% increase in odds of more severe COVID-19 (OR, 3.84; 95% CI, 3.63–4.06); cleft lip/palate, with 187% increase in odds of more severe COVID-19 (OR, 2.87; 95% CI, 2.48–3.32); other classes of digestive system disorders, with 162% increase in odds of more severe COVID-19 (OR, 2.62; 95% CI, 2.42–2.84); respiratory disorders, with 124% increase in odds of more severe COVID-19 (OR, 2.24; 95% CI, 2.04–2.46); neurologic disorders, with 115% increase in odds of more severe COVID-19 (OR, 2.15; 95% CI, 1.99–2.32); musculoskeletal developmental disorders, with 68% increase in odds of more severe COVID-19 (OR, 1.68; 95% CI, 1.58–1.77); chromosomal abnormalities, with 54% increase in odds of more severe COVID-19 (OR, 1.54; 95% CI, 1.43–1.66); genitourinary disorders, with 31% increase in odds of more severe COVID-19 (OR, 1.31; 95% CI, 1.22–1.41); other classes of congenital anomalies, with 23% increase in odds of more severe COVID-19 (OR, 1.23; 95% CI, 1.15–1.32); eye, ear, face, and neck disorders, with 16% increase in odds of more severe COVID-19 (OR, 1.16; 95% CI, 1.03–1.31). A graphical manifestation of the effect of these congenital anomalies on odds of more severe COVID-19 is displayed in Fig. 1.

## Discussion

Congenital circulatory conditions have been shown to be associated with poor COVID-19 outcomes.^8–10,23–26^ There is, however, a dearth of studies and information on COVID-19 severity among patients with other congenital anomalies. The aim of this study was a comprehensive assessment and comparison of predispositions to severe COVID-19 due to congenital anomalies. Considering the large number of individual congenital anomalies, we grouped these conditions by the organ/body systems affected for feasibility and as a guide for investigations into single/specific congenital anomalies. Our findings indicate that all types of congenital anomalies were associated with more severe COVID-19 outcomes in this large pediatric patient cohort, compared to patients without anomalies. We also demonstrated that congenital circulatory conditions (congenital heart defects) conferred a higher risk of severe COVID-19 than any other category of congenital anomaly in this population (as already shown in prior studies^8^), which validates continued focus on SARS-CoV-2 infection prevention and mitigation among patients with congenital circulatory conditions. However, our findings of increased risk for severe COVID-19 in the setting of all types of anomalies indicates a need to extend this prevention and treatment focus to patients with any type of anomaly to prevent severe COVID-19 infection.

Circulatory disorders were closely followed by congenital digestive defects such as cleft lip/palate or malformations of internal digestive organs, in terms of the increased in odds of more severe COVID-19. Previous studies have established a relationship between malnutrition and severe COVID-19.^27,28^ The increased risk of severe COVID-19 in children with congenital digestive conditions may be partly explained by malnutrition attributable to the congenital digestive conditions.

Respiratory and neurologic manifestations of COVID-19 have been clearly established.^29–38^ Cough (56%), rhinorrhea (20%), sore throat (18%), and shortness of breath (12%) are the most common respiratory manifestations of COVID-19 in pediatric patients (Hoang 2020).^39^ The most common neurological manifestations of COVID-19 in children are seizures or status epilepticus (children <5 years old) and anosmia (children >13 years old).^40^ Congenital respiratory anomalies such as congenital pulmonary airway malformation and sequestrations are most often unilateral and do not have a significant effect upon pulmonary mechanics unless very large. Pulmonary hypoplasia is most often observed in the setting of cardiac anomalies or congenital diaphragmatic hernia. However, congenital respiratory anomalies were shown in our data to be associated with a higher risk of severe COVID-19. Our search of the literature on this topic was limited by a paucity of published data on respiratory anomalies and COVID-19 outcomes in pediatric and adult patients. In addition, patients with preexisting/chronic respiratory or neurologic conditions have been shown to have poor COVID-19 outcomes.^8^ Consequently, preexisting congenital defects in these systems are likely to result in more severe COVID-19 outcomes, and our findings confirmed this.

All other congenital anomalies, including those affecting the musculoskeletal and genitourinary systems and chromosomal abnormalities, were associated with significant increases in the odds of more severe COVID-19. This implies that there may be additional physiologic burdens from congenital defects that predispose these patients to worse COVID-19 outcomes. The increased odds of more severe COVID-19 among children with congenital anomalies follows a similar pattern to respiratory syncytial virus (RSV) infection. Incomplete development and malformations of critical organs are associated with worse RSV outcomes as well.^41–45^ Further studies are needed to clarify the causal pathways between congenital anomalies and increased risk of severe COVID-19.^46^ It is important to note that patients with congenital anomalies may be at higher risk for additional comorbid/chronic conditions that have been associated with higher risk of severe outcomes of COVID-19. Consequently, we accounted for related conditions already known to be associated with severe outcomes.^47–50^

Prior research has demonstrated that children with CHD are at higher risk for severe respiratory infections than non-CHD patients due to physiological factors such as: increased pulmonary blood flow; increased pulmonary venous pressures related to poor ventricular function, leading to pulmonary edema, and therefore decreased functional residual capacity; and higher predisposition to hypoxia due to ventilation-perfusion mismatch and/or the anatomical nature of the CHD.^51,52^ Both adults and children with COVID-19 infections and CHD have been shown to have longer length of stay and more complications than patients without CHD, while children with CHD have been shown to have higher mortality as well.^53^ Our current study findings are in line with these prior studies and others.

This study had several limitations. Data related to the severity of underlying congenital defects was not available and may be a source of bias. Data on diagnoses in the database was limited to information from 2015. Consequently, only children diagnosed or treated for a congenital defect from 2015 were captured as having a history of such defects, leading to the exclusion of those with conditions that resolved prior to 2015. Cases of the multisystem inflammatory syndrome in children (MIS-C) were not excluded due to late introduction of the corresponding diagnosis code and complexity of the corresponding definition. Inpatient deaths in this cohort were assumed to be due to COVID-19 because it was not possible to determine which deaths were due to other causes. Data on clinic visits and children infected at home were not captured, and results are therefore conditional on seeking hospital or emergency department care and are thus limited to the more severely ill children. The health systems considered included all census regions of the US, but local outbreaks may have been masked at the census region level. The use of demographic variables for matching and modeling was to reduce associated bias and may not generalize to the entire pediatric population. Studies dedicated to assessing related impacts may be required to provide more robust estimates of their effects. Lastly, SARS-CoV-2 variants were not captured in the database, and there may be other unmeasured confounders with temporal dependence that arose as the pandemic evolved. To reduce bias due to these unmeasured confounders, we matched patients on month of encounter. Furthermore, as part of de-identification procedures, all dates were consistently shifted by 35 days at most in either direction. Consequently, months of SARS-CoV-2 infection used for matching were within one month of each other if not exact.

## Conclusion

All congenital anomalies examined in this study were found to be associated with increased COVID-19 severity in pediatric patients in the US. The results presented in this study highlight the necessity for more studies into specific anomalies and for pediatric health care professionals to offer timely intervention and care to pediatric patients with both congenital anomalies and SARS-CoV-2 infection.

## Supplementary information

Supplemental Materials

## Data Availability

The data that support the findings of this study are available from Oracle® Health but restrictions apply to the availability of these data, which were used under a data use agreement for the current study, and so are not publicly available. Data are however available from the authors upon reasonable request and with permission of Oracle® Health.
