# Supplementary material for: Congenital anomalies and predisposition to severe COVID-19 among pediatric patients in the United States
Source: Pediatr Res. 2024 Feb 16;96(3):792–8. doi: 10.1038/s41390-024-03076-9 (PMC11499254; doi:10.1038/s41390-024-03076-9)
Supplement: Supplementary file 1 — Supplemental Materials [file 41390_2024_3076_MOESM1_ESM.pdf]

Supplemental Materials

Table S1: Summary statistics, by COVID-19 severity, on full data before propensity score matching

	COVID-19 Severity*		
**Variables**	Mild<br>(n = 85,649)	Moderate<br>(n = 14,317)	Severe<br>(n = 7,014)
Age (median, IQR)	2 (0, 9)	4 (1, 12)	2 (0, 11)
Sex			
Female	415,956 (49%)	26,243 (44%)	9,229 (44%)
Male	429,151 (51%)	33,908 (56%)	11,461 (55%)
Unknown	1,651 (0.2%)	148 (0.2%)	58 (0.3%)
Race			
White	522,127 (62%)	36,901 (61%)	11,556 (56%)
American Indian/Alaska Native	7,749 (0.9%)	1,029 (1.7%)	241 (1.2%)
Asian or Pacific Islander	16,514 (2.0%)	1,698 (2.8%)	648 (3.1%)
Black or African American	165,120 (20%)	10,118 (17%)	4,055 (20%)
Mixed racial group	7,045 (0.8%)	703 (1.2%)	145 (0.7%)
Other racial group	78,129 (9.2%)	5,770 (9.6%)	2,297 (11%)
Unknown racial group	50,074 (5.9%)	4,080 (6.8%)	1,806 (8.7%)
Payor			
Commercial	221,519 (26%)	19,332 (32%)	6,081 (29%)
Governmental	275,217 (33%)	21,968 (36%)	6,402 (31%)
Other or unknown	334,934 (40%)	18,119 (30%)	8,048 (39%)
Self-Pay	15,088 (1.8%)	880 (1.5%)	217 (1.0%)
Encounter type			
Admitted for observation	38,258 (4.5%)	14,856 (25%)	1,333 (6.4%)
Emergency	547,536 (65%)	10,323 (17%)	1,270 (6.1%)
Inpatient	69,914 (8.3%)	34,593 (57%)	18,145 (87%)
Urgent care encounter	191,050 (23%)	527 (0.9%)	0 (0%)
Obesity	108,769 (13%)	10,469 (17%)	3,514 (17%)
Non-congenital chronic conditions (Median, IQR)	0 (0, 0)	0 (0, 1)	1 (0, 3)
**Congenital anomalies by body system**			
Neurologic	6,824 (0.8%)	3,026 (5.0%)	2,547 (12%)
Eye, ear, face, and neck	2,854 (0.3%)	717 (1.2%)	698 (3.4%)
Circulatory	13,017 (1.5%)	4,658 (7.7%)	4,606 (22%)
Respiratory	3,901 (0.5%)	1,659 (2.8%)	1,808 (8.7%)
Cleft lip/palate	1,087 (0.1%)	531 (0.9%)	402 (1.9%)
Other digestive systems	4,796 (0.6%)	2,097 (3.5%)	1,170 (5.6%)
Genitourinary	8,303 (1.0%)	2,300 (3.8%)	1,387 (6.7%)
Musculoskeletal	13,612 (1.6%)	3,976 (6.6%)	2,899 (14%)
Other congenital anomalies	8,404 (1.0%)	2,519 (4.2%)	2,034 (9.8%)
Chromosomal	7,261 (0.9%)	2,512 (4.2%)	2,088 (10%)
**Medications**			

Remdesivir	192 (<0.1%)	628 (1.0%)	651 (3.1%)
COVID-19 convalescent plasma	10 (<0.1%)	12 (<0.1%)	11 (<0.1%)
Dexamethasone	54,785 (6.5%)	24,937 (41%)	8,286 (40%)
Heparin	2699 (0.3%)	3392 (5.6%)	4781 (23.0%)
Immunoglobin therapy	1,056 (0.1%)	368 (0.6%)	369 (1.8%)
Methylprednisolone	4,613 (0.5%)	4,579 (7.6%)	3,756 (18%)
Rituximab	210 (<0.1%)	113 (0.2%)	42 (0.2%)
Tocilizumab	37 (<0.1%)	45 (<0.1%)	73 (0.4%)
Aspirin	2,628 (0.3%)	1,750 (2.9%)	1,810 (8.7%)
Lopinavir/ritonavir	2 (<0.1%)	1 (<0.1%)	1 (<0.1%)
